# Supplementary material for: A Dark Septate Endophyte Improves Cadmium Tolerance of Maize by Modifying Root Morphology and Promoting Cadmium Binding to the Cell Wall and Phosphate
Source: J Fungi (Basel). 2023 Apr 29;9(5):531. doi: 10.3390/jof9050531 (PMC10219085; doi:10.3390/jof9050531)
Supplement: Supplementary file 1 [file jof-09-00531-s001.zip › jof-2221723-supplementary.pdf]

Supplementary Materials

Table S1. DSE infection characteristics in maize roots

Cd stress (mg·kg^{-1})	Treatment	Colonization rate (%)
0	CK	—
	DSE	25.8±2.36 c
5	CK	—
	DSE	30.0±6.50 b
10	CK	—
	DSE	35.0±5.1 ab
20	CK	—
	DSE	42.6±4.1 a

All values represent the means ± standard deviations, n=4. Cd: cadmium treatment, CK: the control of non-inoculation, DSE: *Exophiala pisciphila* inoculation. Different little letters in the table refer to $p < 0.05$ according to the LSD test. "—" indicates the index of the material was not detected.

Table S2. Correlation analysis between plant biomass, root morphology and chemical forms, subcellular distribution

	Cd content	Biomass	Root length	Root surface area	Average root diameter	Root tips number	Root branch number	Root crossing number
**Cd Content**	1	-0.449**	0.371**	0.025	-0.381**	0.594**	0.319*	0.374**
**Inorganic Cd**	0.589**	-0.399**	0.004	-0.167	-0.117	0.168	-0.019	0.001
**Water soluble Cd**	0.604**	-0.340*	0.133	-0.063	-0.143	0.366*	0.075	0.053
**Pectates and protein integrated Cd**	0.811**	-0.303*	0.366*	0.190	-0.448**	0.785**	0.190	0.065
**Insoluble phosphate Cd**	0.569**	0.054	0.580**	0.047	-0.319*	0.510**	0.399*	0.505**
**Oxalate Cd**	0.595**	-0.560**	0.208	-0.050	-0.302*	0.389**	0.218	0.237
**Cell wall**	0.850**	-0.117	0.621**	0.146	-0.528**	0.796**	0.538**	0.546**
**Soluble fractions**	0.830**	-0.127	0.552**	0.116	-0.475**	0.615**	0.352*	0.407**
**Organelle**	0.882**	-0.082	0.575**	0.166	-0.463*	0.616**	0.477**	0.331**

"*" means significant difference (p <0.05), and "**" means very significant difference (p <0.01), respectively. n=24.
